# Characterization of the insulin sensitivity of ghrelin receptor KO mice using glycemic clamps

**DOI:** 10.1186/1472-6793-11-1

**Published:** 2011-01-06

**Authors:** Yong Qi, Kenneth A Longo, Derek J Giuliana, Samantha Gagne, Tom McDonagh, Elizabeth Govek, Anna Nolan, Chaoseng Zou, Kristen Morgan, Jeffrey Hixon, Jeffrey O Saunders, Peter S DiStefano, Brad J Geddes

**Affiliations:** 1 Elixir Pharmaceuticals, Inc., 12 Emily St., Cambridge, MA 02139, USA

## Abstract

**Background:**

We and others have demonstrated previously that ghrelin receptor (*GhrR*) knock out (KO) mice fed a high fat diet (HFD) have increased insulin sensitivity and metabolic flexibility relative to WT littermates. A striking feature of the HFD-fed *GhrR *KO mouse is the dramatic decrease in hepatic steatosis. To characterize further the underlying mechanisms of glucose homeostasis in *GhrR *KO mice, we conducted both hyperglycemic (HG) and hyperinsulinemic-euglycemic (HI-E) clamps. Additionally, we investigated tissue glucose uptake and specifically examined liver insulin sensitivity.

**Results:**

Consistent with glucose tolerance-test data, in HG clamp experiments, *GhrR *KO mice showed a reduction in glucose-stimulated insulin release relative to WT littermates. Nevertheless, a robust 1^st ^phase insulin secretion was still achieved, indicating that a healthy β-cell response is maintained. Additionally, *GhrR *KO mice demonstrated both a significantly increased glucose infusion rate and significantly reduced insulin requirement for maintenance of the HG clamp, consistent with their relative insulin sensitivity. In HI-E clamps, both LFD-fed and HFD-fed *GhrR *KO mice showed higher peripheral insulin sensitivity relative to WT littermates as indicated by a significant increase in insulin-stimulated glucose disposal (Rd), and decreased hepatic glucose production (HGP). HFD-fed *GhrR *KO mice showed a marked increase in peripheral tissue glucose uptake in a variety of tissues, including skeletal muscle, brown adipose tissue and white adipose tissue. *GhrR *KO mice fed a HFD also showed a modest, but significant decrease in conversion of pyruvate to glucose, as would be anticipated if these mice displayed increased liver insulin sensitivity. Additionally, the levels of UCP2 and UCP1 were reduced in the liver and BAT, respectively, in *GhrR *KO mice relative to WT mice.

**Conclusions:**

These results indicate that improved glucose homeostasis of *GhrR *KO mice is characterized by robust improvements of glucose disposal in both normal and metabolically challenged states, relative to WT controls. *GhrR *KO mice have an intact 1^st ^phase insulin response but require significantly less insulin for glucose disposal. Our experiments reveal that the insulin sensitivity of *GhrR *KO mice is due to both BW independent and dependent factors. We also provide several lines of evidence that a key feature of the *GhrR *KO mouse is maintenance of hepatic insulin sensitivity during metabolic challenge.

## Background

Administration of exogenous acyl-ghrelin peptide causes insulin resistance in humans [1]. Consistent with this observation, mice with genetic blockade of ghrelin signaling resist diet-induced obesity and show evidence of improved glucose homeostasis under this metabolic stress [2-5]. Pharmacologic antagonism of the ghrelin receptor suppresses appetite, promotes weight loss and improves glucose tolerance [6,7]. Overall, these studies provide compelling evidence that ghrelin and its receptor play an important role in regulation of glucose homeostasis. However, the mechanism[s] linking improved insulin sensitivity and ghrelin signaling are still under investigation.

Circulating ghrelin levels are modulated by changes in nutritional status, such as food deprivation [8] or exposure to HFD [9,10]. Similarly, the physiological consequences of manipulating ghrelin signaling may vary according to metabolic status. For example, Sun et al. [3] demonstrated that ghrelin-deficient mice fed a low-fat diet (LFD) respond to a glucose challenge with improved glucose disposal resulting from increased insulin secretion, relative to WT littermates. In contrast, under conditions of metabolic stress such as high fat diet (HFD)-feeding, ghrelin-deficient mice have improved glucose homeostasis characterized by significantly lower fasting serum insulin levels [5]. Consistent with this latter observation, we have previously observed that HFD-fed *GhrR *KO mice have improved insulin sensitivity corresponding with a striking *reduction *in the insulin required for glucose disposal in response to a glucose challenge [2].

In the current series of experiments we performed hyperglycemic clamps in HFD-fed *GhrR *KO mice in order to examine both insulin sensitivity and the dynamics of insulin secretion in response to a glucose challenge. To further understand the mechanism by which blockade of ghrelin receptor signaling improves insulin sensitivity under conditions of metabolic stress, we also performed a hyperinsulinemic-euglycemic clamp in *GhrR *KO mice fed a HFD and determined tissue glucose dynamics. Our results clearly demonstrate that loss of signaling through the *GhrR *improves insulin sensitivity under conditions of HFD-induced metabolic stress. This insulin sensitivity is characterized by a decreased insulin requirement in the face of a glucose challenge. Additionally, our results reveal a role of hepatic insulin sensitivity in the phenotype of *GhrR *KO mice.

## Results

In preliminary evaluations, *GhrR *KO mice showed improved insulin sensitivity on both low and HFD [2,4]. The fasting blood glucose was 65.5 ± 4.5 vs. 78.4 ± 3.9 mg/dl (p < 0.05) on LFD, and 107.7 ± 3.9 vs. 118.2 ± 7.4 mg/dl on HFD in *GhrR *KO mice and WT mice, respectively (data not shown). The corresponding fasting plasma insulin was 0.66 ± 0.07 vs. 0.77 ± 0.08 ng/ml on LFD, and 0.76 ± 0.09 vs. 1.41 ± 0.32 ng/ml (p < 0.05) on HFD in *GhrR *KO and WT mice, respectively (data not shown).

### Hyperglycemic clamp

**Figure 1:**
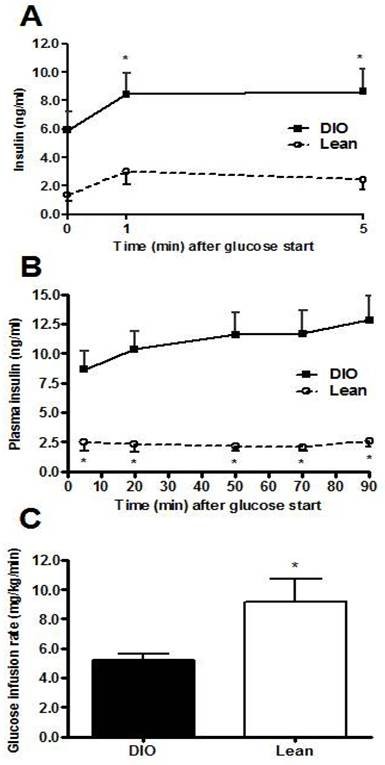
**Hyperglycemic (HG) clamp in anesthetized DIO and lean mice**. The HG clamps were performed over a 90 min period as previously described [29], with the exception of modifications described in the methods section. The hyperglycemic target was 300 mg/dl. Changes in plasma insulin concentration during HG clamp: first phase (A) and second phase (B); glucose infusion rate (C). Data are the means ± SE n = 6-8/group. *p < 0.05 *vs*. lean mice.

**Figure 2:**
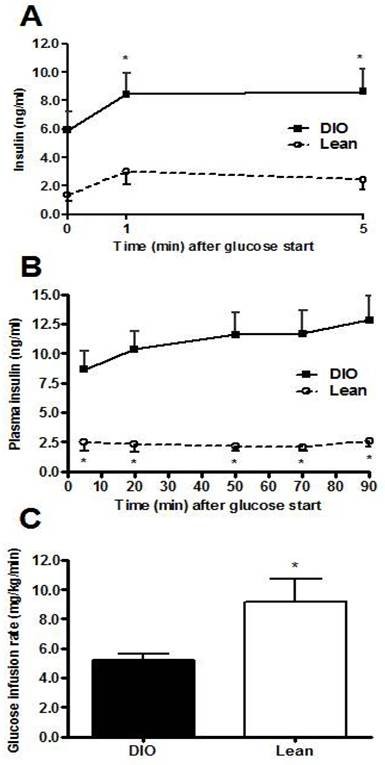
**Hyperglycemic (HG) clamp in anesthetized HFD-fed *GhrR *KO and WT mice**. The HG clamps were performed over a 90 min period as previously described [29], with the exception of modifications described in the methods section. The hyperglycemic target was 300 mg/dl. Changes in plasma insulin concentration during HG clamp: first phase (A) and second phase (B); glucose infusion rate (C). Data are the means ± SE n = 8-10/group. *p < 0.05 *vs*. WT mice.

To evaluate the effects of HFD on islet ß-cell function we performed the HG clamp. This assay allows for the evaluation of glucose-stimulated insulin secretion (GSIS) to an initial glucose challenge. Initial experiments comparing diet-induced obese (DIO) *vs *lean mice confirmed the ability of this assay to reveal the relative insulin resistance of DIO mice relative to lean controls (Figure 1). The pre-study fasted body weight of the DIO mice (45.57 ± 0.96) was significantly greater than the chow-fed littermates (33.46 ± 1.33). Using the identical experimental paradigm, we observed no significant differences in GSIS of *GhrR *KO mice compared with WT animals at either 1 min (2.09 ± 0.41 *vs *1.31 ± 0.15 ng/mL) or 5 min (1.88 ± 0.40 *vs *1.20 ± 0.15 ng/mL) after the initial glucose challenge (Figure 2A). The HG clamp experiments thus highlight that in *GhrR *KO mice the ß-cell function is intact relative to WT controls. However, during the second phase of the experiment WT mice required more insulin to maintain steady state hyperglycemia relative to *GhrR *KO mice, reaching statistical significance at the 70 min (5.72 ± 1.40 *vs *2.41 ± 0.32 ng/mL) and 90 min (6.14 ± 1.50 *vs *2.90 ± 0.34 ng/mL) time points (Figure 2B). Moreover, the GIR was significantly higher in KO mice compared to WT (7.76 ± 1.65 *vs *5.68 ± 1.52 mg/kg/min, Figure 2C), indicative of increased insulin sensitivity of the *GhrR *KO mice. *GhrR *KO mice, therefore, have increased insulin sensitivity characterized by a decreased insulin requirement for glucose disposal relative to WT controls. Prior to initiating the clamp experiment, there was a statistically significant difference in fasted body weight (BW) of *GhrR *KO mice versus WT controls (40.99 ± 1.28 vs 45.07 ± 1.27, respectively). Thus, in this experiment the reduced BW of the *GhrR *KO mice will have contributed to the overall improvement in their insulin sensitivity.

### Hyperinsulinemic-euglycemic clamp

**Figure 3:**
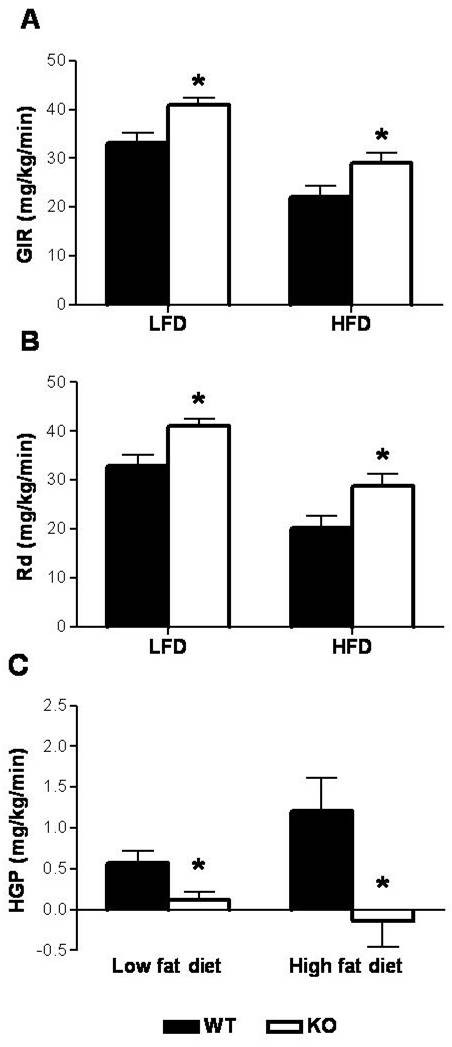
**Hyperinsulinemic-euglycemic clamp in both low and high fat diet *GhrR *KO mice**. (A) Glucose infusion rate. (B) Rate of glucose disposal. (C) Hepatic glucose production. Data are means ± SE from 9-10 mice per group. *, p < 0.05; **, p < 0.01 (*GhrR *KO vs. WT). GIR, glucose infusion rate; Rd, rate of glucose disposal; HPG, hepatic glucose production.

**Figure 4:**
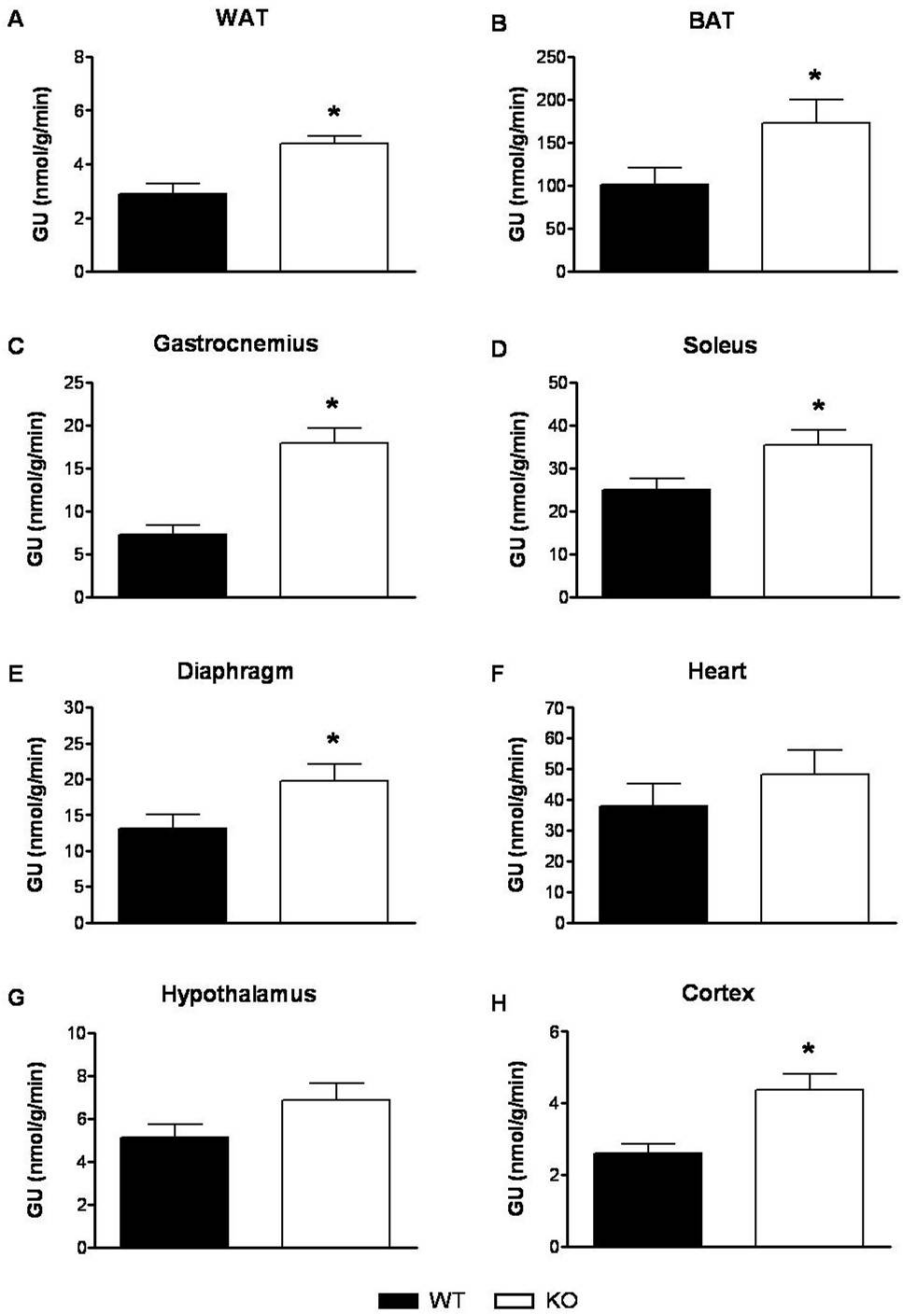
**Tissue glucose uptake in HFD-fed mice of hyperinsulinemic-euglycemic clamp**. At the termination of the HI-clamp, tissues were collected from *GhrR *KO and WT mice (n = 9-10 mice per group) and frozen until analysis. Data are means ± SE. *, p < 0.05 (*GhrR *KO vs. WT). WAT, white adipose tissue; BAT, brown adipose tissue.

We next evaluated hepatic insulin sensitivity and insulin-mediated glucose disposal HFD- and LFD-fed male *GhrR *KO and WT mice (Figure 3). Prior to the study, fasted BW of LFD-fed *GhrR *KO mice (24.86 ± 0.67) was significantly less than WT controls (29.7 ± 1.63). However, in the HFD-fed groups, while there was a trend toward reduced BW in the *GhrR *KO mice (43.90 ± 1.21), it was not statistically significant versus WT (47.91 ± 1.37). Significant increases in the GIR of LFD-fed *GhrR *KO mice (41.0 ± 1.4 mg/kg/min) *vs *WT littermates (33.1 ± 2.1 mg/kg/min) as well as HFD-fed *GhrR *KO mice (29.1 ± 2.1 mg/kg/min) vs WT (22.0 ± 2.3 mg/kg/min) were observed (Figure 3A). Consistent with this, glucose disposal (Rd) was significantly enhanced in *GhrR *KO mice (Figure 3B). HGP (Figure 3C) was significantly reduced in *GhrR *KO vs WT littermates on either a LFD (0.12 ± 0.09 vs. 0.57 ± 0.15 mg/kg/min) or HFD (-0.14 ± 0.32 vs. 1.2 ± 0.41 mg/kg/min). Furthermore, the enhancement of Rd seen in *GhrR *KO mice was associated with significantly increased glucose uptake in 6 out of 8 tissues tested (WAT, BAT, gastrocnemius, soleus, diaphragm and cerebral cortex; see Figure 4). The trend of increased glucose uptake in tissues of *GhrR *KO mice relative to WT controls failed to reach statistical significance for hypothalamus and heart. These results are consistent with the enhanced insulin sensitivity previously observed in conscious HFD-fed *GhrR *KO mice [2]. While differences in BW will have contributed to the insulin sensitivity observed in the LFD-fed KO mice, based on the data obtained in the HFD-fed group it is equally clear that BW is not the only factor involved in the insulin sensitivity of *GhrR *KO mice.

### Increased hepatic insulin sensitivity of conscious *GhrR *KO in a pyruvate tolerance test (PTT)

**Figure 5:**
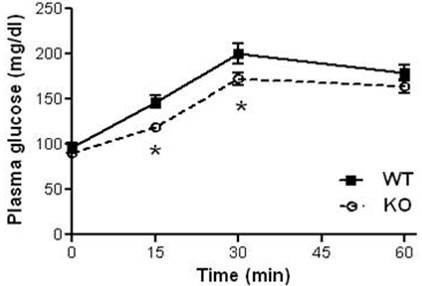
**Pyruvate tolerance test of HFD-fed *GhrR *KO and WT mice**. Overnight fasted HFD-fed *GhrR *KO and WT mice each received an i.p. injection of 1 g/kg sodium pyruvate dissolved in water. Tail vein blood glucose samples were assessed for glucose concentration immediately before injection (time 0) and at the indicated time points post-injection. n = 10 mice per group. Data are means ± SE. *, p < 0.05 (*GhrR *KO vs. WT).

To confirm the reduced HGP of HFD-fed *GhrR *KO mice observed in the clamp study, we conducted a pyruvate tolerance test. There were no significant differences in fasted BW between *GhrR *KO and WT mice fed a HFD (47.63 ± 1.36 and 48.78 ± 1.28, respectively). HFD-fed *GhrR *KO mice had lower plasma glucose levels measured at 15 and 30 minutes after administration of the gluconeogenic substrate pyruvate (Figure 5, p < 0.05), indicative of reduced hepatic glucose output.

### Effects of *GhrR *KO on UCP1 and UCP2 mRNA expression in response to HFD

**Figure 6:**
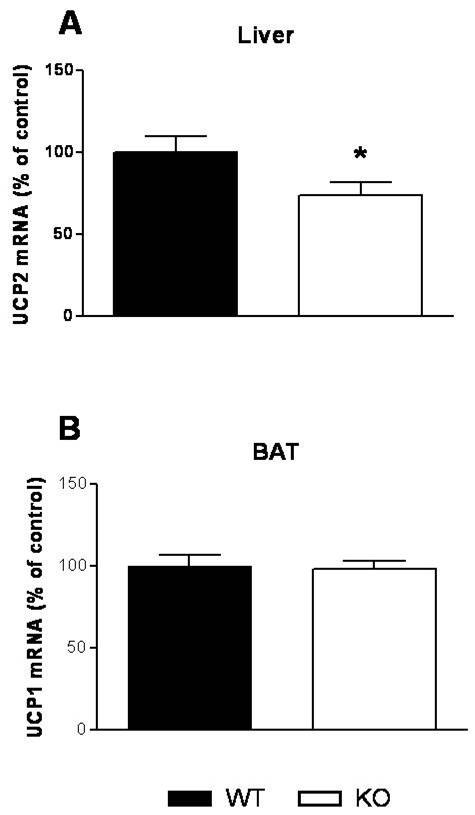
**UCP2 and UCP1 expression levels in liver and BAT, respectively**. (A) mRNA expression of UCP2 of liver in overnight fasted HFD-fed mice. n = 5 mice per group. (B) mRNA expression of UCP1 of BAT in overnight fasted HFD-fed mice. n = 5 mice per group. Data are means ± SE. *p < 0.05 (*GhrR *KO vs. WT). UCP, uncoupling protein.

UCP2 is a fatty acid-responsive mitochondrial inner membrane carrier protein showing wide tissue distribution, but with a substantially increased presence in fatty liver. In comparison with lean animals, hepatic gene expression of UCP2 is increased in *ob*/*ob *mice and DIO rats [11,12]. Increased UCP2 expression in steatotic liver appears to play a reactive and protective role in limiting oxidative damage associated with increased fatty-acid oxidation [13-16]. In the present study *GhrR *KO mice fed a HFD showed significantly reduced UCP2 expression relative to HFD-fed WT mice (Figure 6A), consistent with the decreased hepatic steatosis in HFD-fed *GhrR *KO mice [2].

UCP1 functions in BAT to uncouple substrate oxidation from ATP production leading to the generation of heat [17]. We measured BAT UCP1 expression to determine if the increased BAT glucose uptake may lead to increased thermogenesis. In contrast to liver-UCP2, there was no change in BAT-UCP1 mRNA expression in HFD-fed *GhrR *KO mice (Figure 6B).

## Discussion

Ghrelin and its receptor play an important role in the regulation of glucose homeostasis. Both ghrelin KO and *GhrR *KO mice demonstrate a lower fasting blood glucose with reduced corresponding plasma insulin relative to wild type littermates when fed a HFD [2,5], suggesting that ablation of ghrelin signaling improves insulin sensitivity. In the present studies, we have further characterized the degree and nature of the insulin sensitivity in *GhrR *KO mice in both hyperglycemic and hyperinsulinemic-euglycemic clamp assays. Obviously, decreases in body weight will lead to improvements in glucose homeostasis. However, while there was a tendency for the HFD-fed *GhrR *KO mice to have reduced BW (~5 g) relative to WT controls, this was not always the case. Our lab has evaluated dozens of HFD-fed GhrRKO mouse cohorts over the course of roughly 5 years of experimenting with this model. In so doing we have documented a degree of variability in the BW response to HFD from cohort to cohort such that we occasionally observe non-significant differences between groups. That variability is reflected in the data described in the present work and most likely indicates physiological variability one would expect to see upon repeated testing over the course of a year. However, despite the variability of the BW responses in GhrR KO mouse cohorts, the reported pattern of insulin sensitivity characterized by significantly reduced insulin release coupled with improved glucose disposal is always present. Thus, while not investigated explicitly in these experiments, our data reveal that there is a BW-independent component to the improved insulin sensitivity of *GhrR *KO mice. Further, we provide evidence from HI-clamp, PTT and expression data that suggests an hepatic mechanism for *GhrR *KO insulin sensitivity. Clearly, decreases in BW contribute to the overall improved insulin sensitivity of HFD-fed GhrR KO mice, but it is not the only factor.

Our lab has demonstrated previously that *GhrR *KO mice fed HFD require less insulin for glucose disposal relative to WT controls during a GTT [2]. We therefore conducted HG clamps in order to examine GSIS in these animals in more detail. The *GhrR *KO mice fed a HFD responded to the initial glucose priming dose with a robust first-phase release of insulin that was not significantly different than WT controls, indicating no deficit in their ability to secrete insulin upon challenge [18]. However, a relative trend of decreased insulin requirement of *GhrR *KO mice was maintained throughout the 2^nd ^phase of insulin release [18], and reaching its maximal effect at the point of HG clamp. This pattern is consistent with previous data obtained during a GTT in HFD-fed *GhrR *KO mice (2). Indeed, *GhrR *KO mice fed a HFD are more insulin sensitive compared to WT mice as determined by their significantly increased GIR during the HG clamp. Furthermore *GhrR *KO mice showed decreased HGP, increased glucose disposal and increased tissue glucose uptake in HI-E clamp studies. Remarkably, these effects could be discerned regardless of whether mice were fed a LFD or HFD. In contrast, LFD-fed ghrelin-deficient mice responded to a glucose challenge with increased insulin secretion [3]. The reasons for this apparent dichotomy may be a reflection of differences in islet glucose sensing as a result of chronic exposure to high plasma FFA [19] caused by the HFD. Thus, unlike glucose responsiveness, the underlying improvements of insulin sensitivity resulting from blockade of *GhrR *signaling do not appear to be affected by diet.

In order to understand the mechanism underlying improved hepatic insulin sensitivity, we evaluated hepatic insulin sensitivity of HFD-fed *GhrR *KO mice with a pyruvate tolerance test (PTT), which measures the capacity of the liver to convert pyruvate to glucose, a process that would normally be inhibited by insulin. In comparison to WT control mice, *GhrR *KO mice had a significantly lower gluconeogenic response to the pyruvate challenge, consistent with their relative insulin sensitivity. Therefore, blockade of *GhrR *signaling does not appear to interfere with gluconeogenesis, but rather may decrease HGP via an alternative mechanism, possibly as a secondary result of decreased hepatic lipid burden (2).

Hyperinsulinemia is associated with hepatic steatosis and hyperlipidemia in humans and animal models [20-22], and treatment with insulin-sensitizing drugs ameliorates these conditions [21,23]. Increased fatty acid levels lead to elevations in hepatic UCP2 expression in rats [24], and the circulating free fatty acid concentration correlates with UCP2 expression in white fat and skeletal muscle [25], suggesting that UCP2 is important for the metabolic adaptation of these tissues to excessive fatty acid. Both *GhrR *and *ghrelin *KO mice have improved plasma lipid profiles relative to WT mice on a HFD [2,5]. *GhrR *KO mice have higher fatty acid oxidation and lower lipogenesis, as evidenced by improved hepatic steatosis and lower intestinal triglyceride secretion rate when exposed to HFD [2]. Ghrelin deficiency has been associated previously with a decrease in mitochondrial UCP2 mRNA expression in the livers of chow-fed mice [3]. Likewise, in the present study hepatic expression of UCP2 mRNA was lower in HFD-fed *GhrR *KO mice. Taken together, our results suggest that lower expression of UCP2 mRNA of liver reflects improved lipid metabolism in HFD-fed *GhrR *KO mice and likely contributes to the overall insulin sensitivity in these animals.

Our observation that *GhrR *KO mice had increased glucose uptake into BAT suggested that increased glucose utilization in this tissue may contribute to the improved metabolic phenotype of these mice via increased fatty acid oxidation. BAT is a thermogenic organ in which increased expression of UCP1 decreases mitochondrial energy efficiency, leading to the generation of heat [17]. However, the impact of *GhrR *signaling on thermogenesis is not yet clear. To investigate whether the improved glucose uptake in BAT is associated with increased thermogenesis in *GhrR *KO mice consuming HFD, we measured UCP1 mRNA expression in BAT. We previously reported that *GhrR *deletion in mice had no effect on total energy expenditure [2], which was consistent with the finding that injection of ghrelin had no effect on energy expenditure in rats [8]. In another study, however, central administration of ghrelin suppressed energy expenditure and thermogenesis in BAT via an inhibitory effect on BAT sympathetic nerve activity [26,27]. In the current study, BAT UCP1 mRNA expression was unaffected by genotype in HFD-fed mice, which indicated that diet-induced thermogenesis was not impacted by the loss of ghrelin signaling.

## Conclusions

In summary, these data provide further compelling evidence that blockade of the *GhrR *improves insulin sensitivity. This effect in HFD-fed *GhrR *KO mice has now been demonstrated using the GTT, PTT, HG-clamp and HI-E clamp assays. Furthermore, the characteristic decrease in the insulin required for glucose disposal in *GhrR *KO mice, originally demonstrated in GTT experiments, has been confirmed in HG clamps. The insulin sensitivity of these mice was confirmed by the observation of increased glucose uptake by peripheral tissue during HI-clamp. Thus, we provide evidence for hepatic insulin sensitivity as a contributing factor to the overall insulin sensitivity observed in the *GhrR *KO mice. Whether hepatic insulin sensitivity contributes to, or is secondary to reduced hepatic steatosis was not determined in these studies. *GhrR *KO mice have decreased HGP in both the HI-clamp and PTT. Consistent with these observations we found elevated UCP2 expression levels in the livers of HFD-fed *GhrR *KO mice. These findings demonstrate further the broad metabolic improvements associated with blockade of *GhrR *signaling and substantiate the therapeutic potential of *GhrR *antagonists in the treatment of metabolic diseases, such as type 2 diabetes.

## Methods

### Animals

The *GhrR *KO animals used in these studies were bred from a single founder mouse at the Charles River Laboratories (Wilmington, MA) [28]. *GhrR *+/- mice on the C57BL/6 genetic background (N4) were bred to produce homozygous null and wild type littermate controls. Mice were genotyped as described [2]. Mice were housed in controlled environment rooms (72°F, ~40% humidity, 12 h/12 h in-phase light/dark cycles at 6 am/6 pm) in ventilated racks (Thoren; Hazelton, PA). All mice had *ad libitum *access to normal chow from weaning (PicoLab rodent diet 20, Purina; St. Louis, MO). Diet-induced obesity (DIO) was generated in mice by giving *ad libitum *access to a 60% kcal fat diet (D12492, Research Diets; Rahway, NJ) starting at eight weeks of age, for at least 4 months. In all experiments, pre-study BW was measured the morning after an overnight fast (maximum 16 hours). The work described herein involved the use of several independent cohorts of GhrR KO and WT mice over the course of two years. Experimental procedures were in accordance with regulations of the Elixir Pharmaceuticals Institutional Animal Care and Use Committee.

### Hyperglycemic clamp

The HG clamps were performed over a 90 min period as previously described [29], with the following modifications. During the entire procedure, the animals were kept warm using a heating pad. Tail blood samples (25 μl) were removed at 0 (baseline), 1 and 5 min after a priming intravenous injection of 50% glucose (0.25 g/kg), for the measurement of first phase blood glucose and plasma insulin. Immediately after the 5 min sampling time point animals received an infusion of 20% glucose. Tail blood glucose readings were taken at 5-10 min intervals thereafter and the 20% glucose infusion rate was adjusted in order to reach, and then maintain, blood glucose levels at approximately 300 mg/dl. Twenty-five μl blood samples were collected via the carotid artery at 20, 50, 70 and 90 min after baseline for plasma insulin determination. During the 50-90 min time points, steady state hyperglycemia was established (3 consecutive readings of 300 mg/dl) at which time the GIR was determined. Preliminary experiments were performed comparing diet-induced obese (DIO) vs lean mice prior to conducting experiments on HFD-fed *GhrR *KO vs WT mice (Figure 1).

### Hyperinsulinemic-euglycemic (HI-E) clamp

30-week-old HFD- and LFD-fed male mice were fasted overnight (16-18 h), anesthetized with sodium pentobarbital (50 mg/kg, i.p.), before undergoing a hyperinsulinemic-euglycemic clamp as previously described [30]. During the entire procedure, the animals were kept warm using a heating pad. Thirty min after the right internal jugular vein cannulation, mice underwent the hyperinsulinemic-euglycemic (HI) clamp for 120 minutes. A second dose of anesthetic (10 mg/kg, i.p.) was delivered 30 minutes after the start of the clamp and mice were maintained under anesthesia for the duration of the experiment. A priming dose of human insulin (100 mU/kg, iv; Humulin R; Eli Lilly, Indianapolis, IN) was administered, followed by continuous iv infusion at 50 mU/kg/min. Tail blood samples (4 μl) were collected at 10 min intervals for measurement of glucose (Ascensia Elite glucometer, Bayer, Indianapolis, IN), and 20% glucose was infused to maintain blood glucose between 110 and 140 mg/dl. Insulin-stimulated whole body glucose disposal was assessed using a priming injection of 5-μCi HPLC-purified [3-^3^H] glucose (Perkin Elmer, Boston, MA) followed by continuous infusion at 0.05 μCi/min throughout the study; Once steady state was attained for 30 minutes, a bolus of 10 μCi 2-deoxy-D-[1-^14^C] glucose (Perkin Elmer, Boston, MA) was injected intravenously (minute 75) to determine the insulin-stimulated glucose uptake of various tissues. Insulin was measured in 10 μl blood samples drawn before cannulation surgery and at the end of clamps. The mice were killed at the end of the experiment, and muscles, perigonadal white adipose tissue, and liver were harvested, frozen in liquid nitrogen, and stored at -80°C until processing. The glucose infusion rate (GIR), HGP, rate of glucose disposal (*R*_d_), and tissue glucose uptake were determined as previously described [31,32].

### Pyruvate tolerance test (PTT)

30-week-old, HFD-fed male mice were fasted overnight (16-18 h), before undergoing a PTT as previously described [33]. *GhrR *KO mice and wild type littermates each received an i.p. injection of 1 g/kg sodium pyruvate dissolved in water. Tail-vein blood samples were assessed for glucose immediately before injection (time 0) and at 15, 30 and 60 minutes post-injection.

### RNA preparation and Real time (RT) PCR

Total RNA of flash-frozen liver and BAT from overnight fasted mice was extracted using TRIzol reagent (Invitrogen). The mRNA of PEPCK, G-6-Pase and UCP-2 in liver, and mRNA of UCP-1 in BAT were analyzed by real time PCR and normalized to a housekeeping gene (36B4) (Light Cycler 480 SYBR Green, Roche Diagnostics, Indianapolis, IN).

### Plasma insulin measurement

Plasma insulin was measured by a homogeneous time-resolved fluorescence immunoassay (HTRF) (Cisbio-US, Inc, Bedford, MA).

### Statistical analyses of data

All values are expressed as the mean ± SE. Changes in various parameters were analyzed by two-way *ANOVA *and pair-wise differences assessed using Bonferroni post-hoc tests (GraphPad Prism, San Diego, CA). We compared end-of-study values using the Student's *t-*test. *P *< 0.05 was considered significant.

## Authors' contributions

YQ designed and conducted HG and HI-E clamps and PTT assays and assisted in the writing/editing of the manuscript. KAL assisted in conducting clamp and PTT assays and in writing/editing the manuscript. TM and AN assisted in conducting PTT and expression assays. EG assisted in conducting PTT and expression assays as well as writing/editing the manuscript. DJG, SG, CZ, KM, and JH assisted in conducting clamp and PTT assays. JOS assisted in writing/editing manuscript. PSD was involved in study design and assisted in writing/editing manuscript. BJG was involved in study design and wrote the manuscript. All authors read and approved the final manuscript.

## References

[B1] Vestergaard ET Djurhuus CB Gjedsted J Nielsen S Moller N Acute effects of ghrelin administration on glucose and lipid metabolism J Clin Endocrinol Metab 2008 93 438 444 10.1210/jc.2007-2018 18042651

[B2] Longo KA Charoenthongtrakul S Giuliana DJ Govek EK McDonagh T Improved insulin sensitivity and metabolic flexibility in ghrelin receptor knockout mice Regul Pept 2008 18453014 10.1016/j.regpep.2008.03.011

[B3] Sun Y Asnicar M Saha PK Chan L Smith RG Ablation of ghrelin improves the diabetic but not obese phenotype of ob/ob mice Cell Metab 2006 3 379 386 10.1016/j.cmet.2006.04.004 16679295

[B4] Zigman JM Nakano Y Coppari R Balthasar N Marcus JN Mice lacking ghrelin receptors resist the development of diet-induced obesity J Clin Invest 2005 115 3564 3572 10.1172/JCI26002 16322794 PMC1297251

[B5] Wortley KE del Rincon JP Murray JD Garcia K Iida K Absence of ghrelin protects against early-onset obesity J Clin Invest 2005 115 3573 3578 10.1172/JCI26003 16322795 PMC1297252

[B6] Rudolph J Esler WP O'Connor S Coish PD Wickens PL Quinazolinone derivatives as orally available ghrelin receptor antagonists for the treatment of diabetes and obesity J Med Chem 2007 50 5202 5216 10.1021/jm070071+ 17887659

[B7] Esler WP Rudolph J Claus TH Tang W Barucci N Small-molecule ghrelin receptor antagonists improve glucose tolerance, suppress appetite, and promote weight loss Endocrinology 2007 148 5175 5185 10.1210/en.2007-0239 17656463

[B8] Tschop M Smiley DL Heiman ML Ghrelin induces adiposity in rodents Nature 2000 407 908 913 10.1038/35038090 11057670

[B9] Lee HM Wang G Englander EW Kojima M Greeley GH Jr Ghrelin, a new gastrointestinal endocrine peptide that stimulates insulin secretion: enteric distribution, ontogeny, influence of endocrine, and dietary manipulations Endocrinology 2002 143 185 190 10.1210/en.143.1.185 11751608

[B10] Moesgaard SG Ahren B Carr RD Gram DX Brand CL Effects of high-fat feeding and fasting on ghrelin expression in the mouse stomach Regul Pept 2004 120 261 267 10.1016/j.regpep.2004.03.018 15177945

[B11] Chavin KD Yang S Lin HZ Chatham J Chacko VP Obesity induces expression of uncoupling protein-2 in hepatocytes and promotes liver ATP depletion J Biol Chem 1999 274 5692 5700 10.1074/jbc.274.9.5692 10026188

[B12] Fan JG Ding XD Wang GL Xu ZJ Tian LY [Expression of uncoupling protein 2 and its relationship to the content of adenosine triphosphate in the nonalcoholic fatty livers of rats fed a high-fat diet] Zhonghua Gan Zang Bing Za Zhi 2005 13 374 377 15918975

[B13] Yang S Zhu H Li Y Lin H Gabrielson K Mitochondrial adaptations to obesity-related oxidant stress Arch Biochem Biophys 2000 378 259 268 10.1006/abbi.2000.1829 10860543

[B14] Echtay KS Roussel D St-Pierre J Jekabsons MB Cadenas S Superoxide activates mitochondrial uncoupling proteins Nature 2002 415 96 99 10.1038/415096a 11780125

[B15] Brand MD Esteves TC Physiological functions of the mitochondrial uncoupling proteins UCP2 and UCP3 Cell Metab 2005 2 85 93 10.1016/j.cmet.2005.06.002 16098826

[B16] Serviddio G Sastre J Bellanti F Vina J Vendemiale G Mitochondrial involvement in non-alcoholic steatohepatitis Mol Aspects Med 2008 29 22 35 10.1016/j.mam.2007.09.014 18061659

[B17] Fisler JS Warden CH Uncoupling proteins, dietary fat and the metabolic syndrome Nutr Metab (Lond) 2006 3 38 10.1186/1743-7075-3-38 16968550 PMC1592539

[B18] Nesher R Cerasi E Modeling phasic insulin release: immediate and time-dependent effects of glucose Diabetes 2002 51 Suppl 1 S53 59 10.2337/diabetes.51.2007.S53 11815459

[B19] Cerf ME High fat diet modulation of glucose sensing in the beta-cell Med Sci Monit 2007 13 RA12 17 17179917

[B20] Angulo P Nonalcoholic fatty liver disease N Engl J Med 2002 346 1221 1231 10.1056/NEJMra011775 11961152

[B21] Browning JD Horton JD Molecular mediators of hepatic steatosis and liver injury J Clin Invest 2004 114 147 152 15254578 10.1172/JCI22422 PMC449757

[B22] Nandi A Kitamura Y Kahn CR Accili D Mouse models of insulin resistance Physiol Rev 2004 84 623 647 10.1152/physrev.00032.2003 15044684

[B23] Chavez-Tapia NC Barrientos-Gutierrez T Tellez-Avila FI Sanchez-Avila F Montano-Reyes MA Insulin sensitizers in treatment of nonalcoholic fatty liver disease: Systematic review World J Gastroenterol 2006 12 7826 7831 17203528 10.3748/wjg.v12.i48.7826 PMC4087550

[B24] Armstrong MB Towle HC Polyunsaturated fatty acids stimulate hepatic UCP-2 expression via a PPARalpha-mediated pathway Am J Physiol Endocrinol Metab 2001 281 E1197 1204 11701434 10.1152/ajpendo.2001.281.6.E1197

[B25] Boss O Bobbioni-Harsch E Assimacopoulos-Jeannet F Muzzin P Munger R Uncoupling protein-3 expression in skeletal muscle and free fatty acids in obesity Lancet 1998 351 1933 10.1016/S0140-6736(05)78617-7 9654269

[B26] Yasuda T Masaki T Kakuma T Yoshimatsu H Centrally administered ghrelin suppresses sympathetic nerve activity in brown adipose tissue of rats Neurosci Lett 2003 349 75 78 10.1016/S0304-3940(03)00789-4 12946556

[B27] Tsubone T Masaki T Katsuragi I Tanaka K Kakuma T Ghrelin regulates adiposity in white adipose tissue and UCP1 mRNA expression in brown adipose tissue in mice Regul Pept 2005 130 97 103 10.1016/j.regpep.2005.04.004 15946750

[B28] Sun Y Wang P Zheng H Smith RG Ghrelin stimulation of growth hormone release and appetite is mediated through the growth hormone secretagogue receptor Proc Natl Acad Sci USA 2004 101 4679 4684 10.1073/pnas.0305930101 15070777 PMC384806

[B29] Henquin JC Nenquin M Stiernet P Ahren B In vivo and in vitro glucose-induced biphasic insulin secretion in the mouse: pattern and role of cytoplasmic Ca2+ and amplification signals in beta-cells Diabetes 2006 55 441 451 10.2337/diabetes.55.02.06.db05-1051 16443779

[B30] Cederroth CR Vinciguerra M Gjinovci A Kuhne F Klein M Dietary phytoestrogens activate AMP-activated protein kinase with improvement in lipid and glucose metabolism Diabetes 2008 57 1176 1185 10.2337/db07-0630 18420492

[B31] Kim JK Fillmore JJ Gavrilova O Chao L Higashimori T Differential effects of rosiglitazone on skeletal muscle and liver insulin resistance in A-ZIP/F-1 fatless mice Diabetes 2003 52 1311 1318 10.2337/diabetes.52.6.1311 12765938

[B32] Fisher SJ Kahn CR Insulin signaling is required for insulin's direct and indirect action on hepatic glucose production J Clin Invest 2003 111 463 468 12588884 10.1172/JCI16426 PMC151923

[B33] Gray S Wang B Orihuela Y Hong EG Fisch S Regulation of gluconeogenesis by Kruppel-like factor 15 Cell Metab 2007 5 305 312 10.1016/j.cmet.2007.03.002 17403374 PMC1892530

